# Proangiogenic Growth Factor Therapy for the Treatment of Refractory Angina: A Meta-analysis

**DOI:** 10.1016/j.jscai.2022.100527

**Published:** 2023-01-02

**Authors:** Deshan Weeraman, Daniel A. Jones, Mohsin Hussain, Anne-Marie Beirne, Steven Hadyanto, Krishnaraj S. Rathod, James R. Whiteford, Alice E. Reid, Christos V. Bourantas, Seppo Ylä-Herttuala, Andreas Baumbach, Bernard J. Gersh, Timothy D. Henry, Anthony Mathur

**Affiliations:** a Centre for Cardiovascular Medicine and Devices, William Harvey Research Institute, Queen Mary University of London, London, United Kingdom; b Barts National Institute for Health and Care Research Biomedical Research Centre, Barts Heart Centre & Queen Mary University of London, London, United Kingdom; c Barts Heart Centre, Barts Health National Health Service Trust, London, United Kingdom; d Centre for Microvascular Research, William Harvey Research Institute, Barts & The London Medical School, Queen Mary University of London, London, United Kingdom; e A.I. Virtanen Institute, University of Eastern Finland, Kuopio, Finland; f Department of Cardiovascular Medicine, Mayo Clinic College of Medicine, Rochester, Minnesota; g The Carl and Edyth Lindner Center for Research and Education at The Christ Hospital, Cincinnati, Ohio

**Keywords:** refractory angina, chronic myocardial ischemia, angiogenesis, gene therapy, meta-analysis

## Abstract

**Background:**

Refractory angina (RFA; limiting angina despite optimal medical therapy) is a growing, global problem, with limited treatment options. Therefore, we conducted a systematic review of randomized controlled trials (RCTs) to evaluate the effect of proangiogenic growth factor therapy (in the form of vascular growth factors delivered either as recombinant proteins or gene therapy) in patients with RFA ineligible for revascularization.

**Methods:**

We performed a meta-analysis (PROSPERO: CRD42018107283) of RCTs as per the Preferred Reporting Items for Systematic Reviews and Meta-Analyses methodology. A comprehensive search of the PubMed, CENTRAL, Embase, Cochrane, ClinicalTrials.gov and Google Scholar databases, as well as scientific session abstracts, were performed. The pooled outcomes included major adverse cardiac events (MACE), mortality, myocardial perfusion, and indices of angina severity (Canadian Cardiovascular Society angina class [CCS] and exercise tolerance). A prespecified subgroup analysis was performed for delivery method, vector, and protein type. The standardized mean difference (SMD) or odds ratio (OR) was calculated to assess relevant outcomes. We assessed heterogeneity using the χ^2^ and I^2^ tests.

**Results:**

We included 16 RCTs involving 1607 patients (1052 received proangiogenic growth factor therapy and 555 received a placebo or optimal medical therapy). Our analysis showed a significant decreased risk of MACE (OR, 0.72; 95% confidence interval [CI], 0.55-0.93) and significantly improved CCS class (SMD, −0.55; 95% CI, −1.10 to 0.00), but not mortality (OR, 0.66; 95% CI, 0.28-1.54) or exercise tolerance (SMD, 0.47; 95% CI, −0.14 to 1.09), in treated patients compared to those in the control group.

**Conclusions:**

Proangiogenic growth factor therapy is a promising treatment option for RFA, with beneficial effects seen on MACE and CCS class. The results of ongoing trials are needed before it can be considered for clinical practice.

## Highlights

• Further therapeutic options are needed for the treatment of refractory angina.
• Proangiogenic growth factor therapy led to improvements in major adverse cardiac events and Canadian Cardiovascular Society class.
• Larger studies are justified to look for a definitive beneficial signal.
• The optimal delivery route, delivery vector, and protein type are yet to be identified.

## Central Illustration

**Figure:**
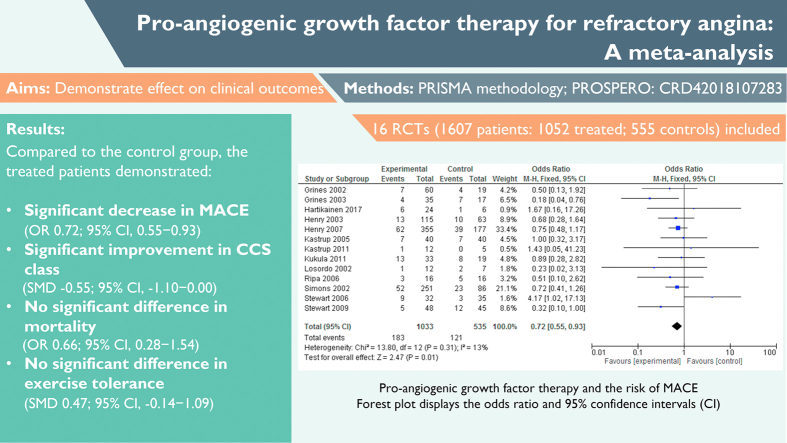

## Introduction

Refractory angina (RFA) is characterized by chronic chest pain (duration ≥ 3 months) in the presence of coronary artery disease that persists despite optimal medical, interventional, and surgical management.^1^ The global prevalence of RFA is increasing; in Europe, there are 30,000 to 50,000 new cases per year, and it affects 600,000 to 1,800,000 people in the United States, with ∼50,000 new cases per year.^2^ The management of angina using conventional treatment strategies remains challenging; a significant number of patients remain symptomatic despite percutaneous coronary intervention, coronary artery bypass graft surgery, and antianginal drugs. It costs $33,000 per year to treat a patient with RFA in the United States,^3^ and studies have suggested that there are further additional expenses due to productivity losses (indirect costs)^4^ and recurrent admissions.^5^

Refractory angina is linked to increased cardiovascular morbidity and mortality. In the international CLARIFY study (N = 32,105), 20% of patients with stable coronary artery disease had anginal symptoms, and these were associated with worse cardiovascular clinical outcomes over the 2 years of follow-up.^6^ Although the long-term survival of patients with RFA and obstructive coronary artery disease is better than previously thought, in the OPTIMIST registry (N = 1200), 20% of the patients died during a median follow-up duration of 5 years, mainly from cardiovascular causes (the mortality rate was 3.9% at 1 year and 28.4% at 9 years), and anginal symptoms were associated with increased adverse cardiac events.^7^^,^^8^ Although the overall mortality rate is low, estimated at ∼3% to 4% per year,^7^ RFA has profound effects on the quality of life of affected patients. Patients experience severe chest pain, resulting in low levels of health-related quality of life and multiple hospitalizations.^8^

Refractory angina is a multifactorial process, of which ischemia is an important driver.^8^ Therefore, any therapy that can stimulate vascular growth could provide potential therapeutic benefit. The direct transfer of angiogenic vascular growth factors to ischemic myocardium stimulates angiogenesis and enhances collateral blood supply, thereby reducing the ischemic burden.^9^

Promising preclinical data^10^ have led to clinical trials of proangiogenic growth factor therapy (either recombinant protein or gene therapy) as a treatment option for RFA. These trials used proteins from the vascular endothelial growth factor (VEGF; a dimeric glycoprotein that regulates endothelial cell behavior, angiogenesis, and lymphangiogenesis) and fibroblast growth factor (FGF; a pluripotent growth factor capable of stimulating growth and promoting vascular tree branching) families.

To date, meta-analyses have studied VEGF and gene therapy for ischemic coronary artery disease^11^ and cell therapy for RFA.^12^ However, there has been no definitive assessment of the efficacy of proangiogenic growth factor therapy (defined as either recombinant protein or gene therapy) for RFA assessing all vectors and delivery methods. Therefore, we conducted this comprehensive meta-analysis to assess the safety and impact of proangiogenic growth factor therapy on clinical outcomes (mortality and major adverse cardiac events [MACE]), measures of angina symptoms (Canadian Cardiovascular Society [CCS] class and total exercise time), and myocardial perfusion.

## Methods

### Data sources and search strategy

The study protocol was registered prospectively with the PROSPERO international registry (CRD42018107283), and the systematic review was conducted following the Preferred Reporting Items for Systematic Reviews and Meta-analyses (PRISMA) guidelines.^13^ An experienced medical reference librarian performed the search strategy and subsequent digital literature review. The National Institute for Health and Care Excellence’s Healthcare Database Advanced Search tool (https://hdas.nice.org.uk/) searched the MEDLINE, Google Scholar, and Embase databases using a sensitive strategy, with text searches and subject headings used in conjunction. The keywords using Medical Subject Headings or Embase subject headings terms included “refractory angina,” “chronic myocardial ischaemia,” “coronary artery disease,” “drug-resistant angina,” “gene therapy,” and “angiogenic proteins.” The results were limited to clinical trials, reviews, or meta-analyses, with no date or language limitations. The search included studies published until February 2, 2022. We also manually searched for further potential citations from eligible articles. The results were deduplicated using the Mendeley software and manually screened. The complete search strategy is available in the Supplementary Material. Where needed, we contacted the authors for further data (described in “Dealing with missing data”). Ethical approval was not required because this was a meta-analysis of existing clinical trials (all of which independently received ethical approval).

### Study selection

Studies were eligible if they met the following criteria: were full-length publications in peer-reviewed journals, evaluated the use of proangiogenic growth factor therapy, included patients with no further revascularization options, included patients with ongoing stable angina (equivalent to CCS class II-IV) despite optimal medical therapy, and were randomized controlled trials (RCTs). We excluded studies that used proangiogenic growth factor therapy at the time of transmyocardial or coronary revascularization given that this could independently affect the outcome and confound the effect of the angiogenic protein. We also excluded trials that purely assessed safety as an outcome without any efficacy data. One trial used a historical control group^14^ from an RCT that was performed by the same study team and used identical inclusion criteria.

### Data synthesis and extraction

The data were prespecified before the literature search. The extracted study information included the following: study design; inclusion or exclusion criteria; vector type (if applicable); baseline demographics, medical history, or medications; myocardial perfusion; use of antianginal medications; CCS class; total exercise time; MACE; and mortality. Three investigators (D.W., D.J. and M.H.) independently assessed the studies’ eligibility for inclusion. From the included studies, 2 reviewers (D.W. and D.J.) extracted the aforementioned information.

To understand the clinical benefit of angiogenic protein therapy, our primary endpoints were all-cause mortality and MACE (defined as myocardial infarction, cardiac-related hospitalization, and mortality). Individually reported events from each trial were combined into the prespecified composite MACE endpoint. We also examined the measures of angina severity (myocardial perfusion, CCS class, use of antianginal medications, quality-of-life measurements, and exercise tolerance). We performed a subgroup analysis for delivery route, vector, and protein type.

### Statistical analysis

We calculated odds ratios (OR) for safety, all-cause mortality, and MACE. Continuous data were reported either as mean (standard deviation) or median (interquartile range). When the standard deviation was unavailable from the data, the Cochrane Handbook was used to calculate variance.^15^ For continuous variables (CCS class, exercise tolerance, and use of antianginal medications), we used standardized mean difference (SMD) to compare the mean change in the variable at the endpoint between the 2 groups and the absolute change in the variable within the group. A visual assessment of forest plots and the I^2^ statistic were used to assess heterogeneity among studies. For mortality, we detected no significant heterogeneity among studies (I^2^ = 0%), and no difference in effect was seen following a sensitivity analysis. For MACE, we detected no significant heterogeneity among studies (I^2^ = 13%), and no difference in effect was seen following a sensitivity analysis. The meta-analyses were performed using a fixed-effects model or a random-effects model if heterogeneity was encountered.

### Quality assessment

**Figure 1:**
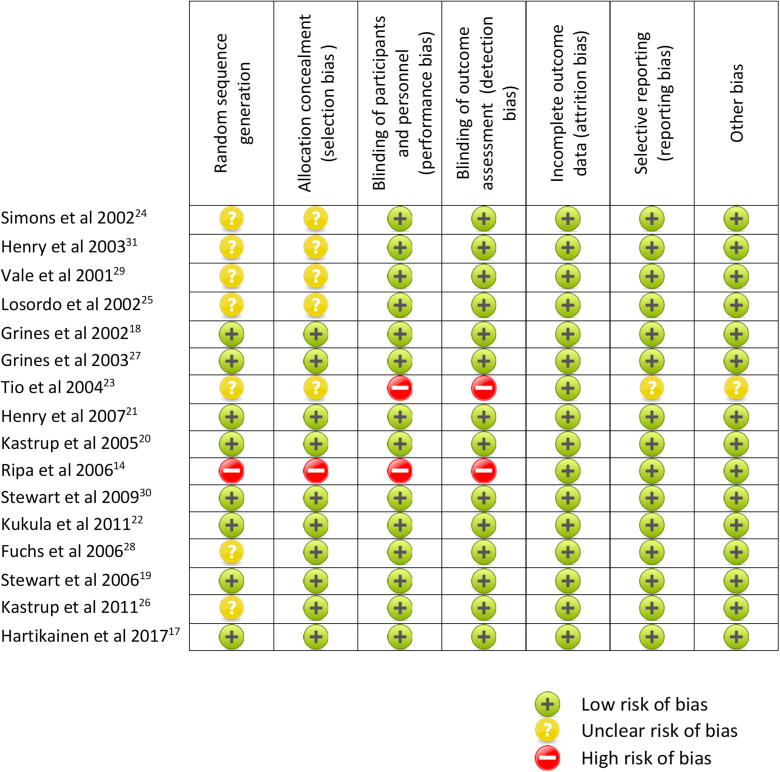
Risk-of-bias assessment.

A group of reviewers (D.W., M.H., and A.M.B.) independently assessed the methodologic quality of selected studies using the Cochrane risk of bias tool.^16^ We considered each study’s risk of bias by assessing random sequence generation, the method of allocation concealment, blinding of participants and personnel (performance bias), blinding of outcome assessment (detection bias), incomplete outcome data (attrition bias), selective reporting (reporting bias) and other biases (Figure 1). We used https://clinicaltrials.gov/ to determine whether there were discrepancies between the planned and reported analyses. Funnel plots for the risk of publication bias are presented in Supplemental Figure S1.

## Results

### Identified and eligible studies

**Figure 2:**
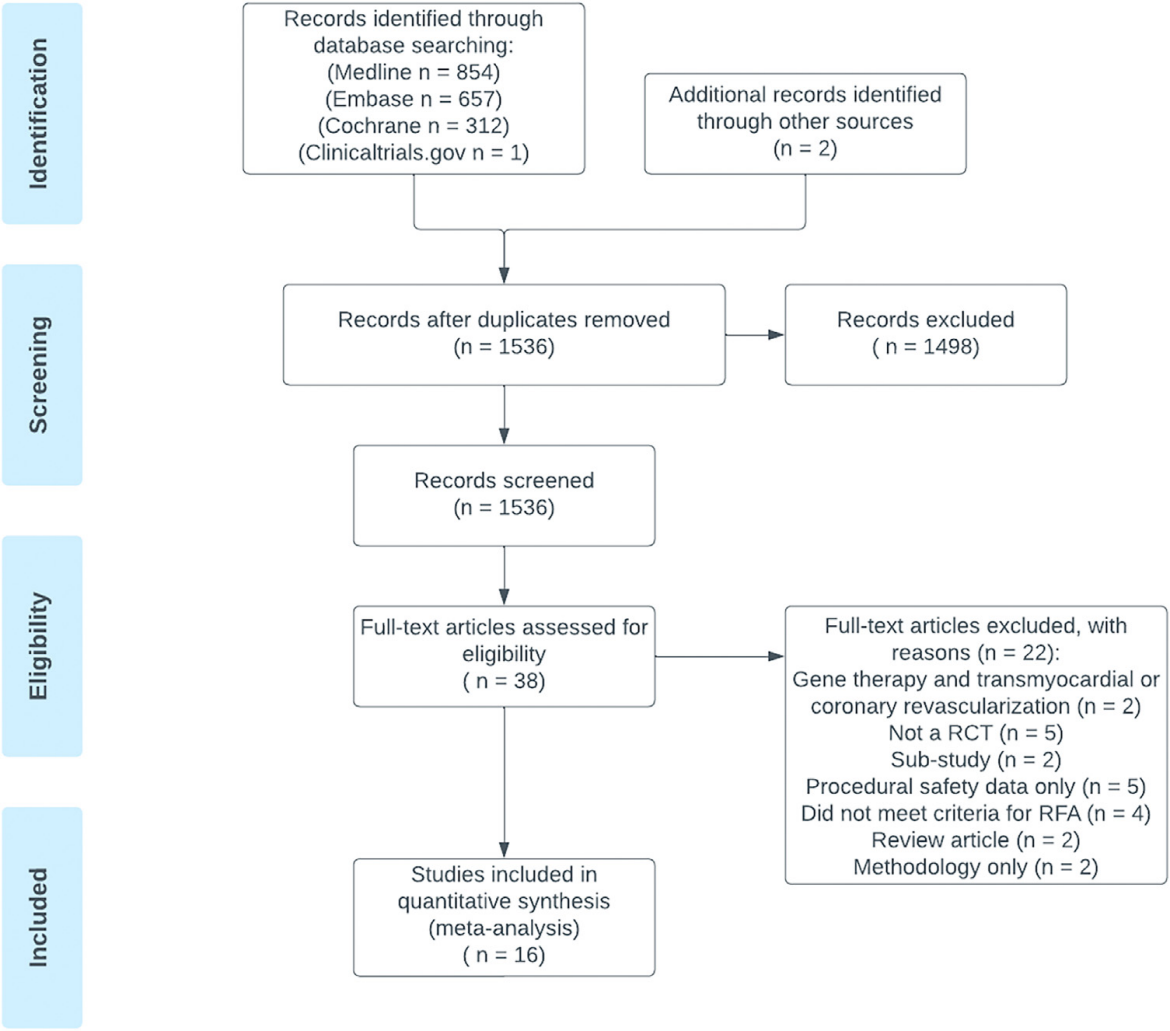
**Study flow chart: Preferred Reporting Items for Systematic Reviews and Meta-Analyses flow diagram illustrating the study selection process for the meta-analysis.** RCT, randomized controlled trial; RFA, refractory angina.

The search strategy identified 1536 potential articles (Figure 2). After removing duplicates, we screened their titles and abstracts. Of these, we selected 38 for full-text review. Ultimately, 16 RCTs satisfied all the inclusion criteria.^14^^,^^17^, ^18^, ^19^, ^20^, ^21^, ^22^, ^23^, ^24^, ^25^, ^26^, ^27^, ^28^, ^29^, ^30^, ^31^ One study reported outcomes with 2 different doses of protein-based –therapy.^31^ We combined the treated patients for analysis. Another study assessed 2 different treatment options (laser direct myocardial revascularization and gene therapy VEGF_165_); only patients treated with proangiogenic growth factor therapy were included.^23^

### Characteristics of included studies

Sixteen RCTs, with a total of 1607 patients (1052 patients underwent proangiogenic vascular growth therapy and 555 patients were managed with a placebo or optimal medical therapy) were included (Supplemental Table S1). The studies occurred between 2001 and 2017 and consisted of 12 double-blind RCTs,^17^^,^^18^^,^^20^, ^21^, ^22^^,^^24^, ^25^, ^26^, ^27^, ^28^^,^^30^^,^^31^ 1 single-blind RCT,^29^ 2 open-label RCTs,^19^^,^^23^ and 1 single-arm trial that used a historical control group.^14^

Two trials used recombinant proteins,^24^^,^^31^ and 14 trials used genes to deliver proangiogenic growth factor therapy.^14^^,^^17^, ^18^, ^19^, ^20^, ^21^, ^22^, ^23^^,^^25^, ^26^, ^27^, ^28^, ^29^, ^30^ All the trials used VEGF or FGF: 1 (VIF-CAD) used a combination of both^22^ and 1 (NEUOPEGEN) used VEGF in combination with granulocyte colony-stimulating factor.^14^ Two forms of FGF were used: FGF-2^24^ and FGF-4.^18^^,^^21^^,^^27^ Six isoforms of VEGF were used: VEGF,^31^ VEGF-2,^25^^,^^29^ VEGF_121_,^28^ VEGF-A_121_,^19^^,^^26^ VEGF-A_165_,^14^^,^^20^^,^^22^^,^^23^^,^^30^ and VEGF-D^ΔNΔC^.^17^ Four trials used an intracoronary infusion,^18^^,^^21^^,^^24^^,^^27^ 1 combined an intracoronary infusion with a peripheral intravenous infusion,^31^ 1 used a minithoracotomy^19^ and 10 used a NOGA (Biosense Webster) guided intramyocardial injection.^14^^,^^17^, ^18^, ^20^^,^^22^^,^^23^^,^^25^^,^^26^^,^^28^^,^^30^ The trials’ follow-up duration ranged from 3 to 24 months.

### Dealing with missing data

When needed, we contacted authors for additional information. Additional data were obtained for some trials^18^^,^^19^^,^^27^^,^^30^; however, we were unable to obtain it for others despite our attempts.^23^, ^24^, ^25^, ^26^^,^^28^, ^29^^,^^31^ In some cases, data were no longer available.^18^^,^^21^ Any missing data were not imputed.

### Risk of bias in included studies

Two trials were deemed to have a high risk of bias (Figure 1). One trial was determined to have a high risk of random sequence generation, selection, performance, and detection bias because it was a single-arm, open-label study with a historical control group.^14^ One trial was deemed to have a high risk of performance and detection biases because the participants and personnel were not blinded to the treatment or outcome.^23^

When known, the risk of bias in the other 14 studies was determined to be low because they were of sound methodologic quality. The risk of publication bias was assessed using funnel plots (Supplemental Figure S1) and found to be low. There was good agreement between the reviewers for study inclusion, data abstraction and quality assessment; any disagreements were resolved by a fourth reviewer (D.J.).

### Primary outcomes

#### Mortality

**Figure 3:**
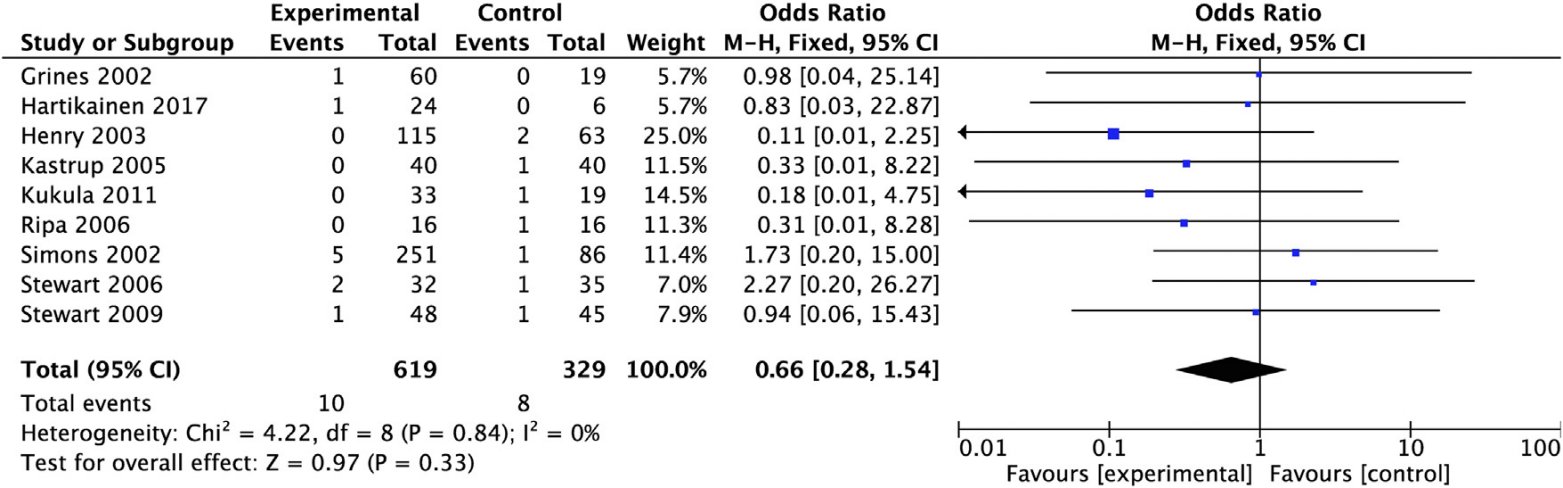
**Proangiogenic growth factor therapy and risk of mortality.** Forest plot displays the odds ratio (OR) and 95% CI. df, degrees of freedom; M-H, Mantel-Haenszel.

The analysis included all-cause mortality outcomes from 14 trials. Two trials were excluded: 1 reported no mortality data^23^ and 1 combined MACE and mortality.^21^ The analysis demonstrated a mortality of 2% (8/365) in the control group and 1% (10/687) in the treatment group. There was a nonsignificant between-group reduction (OR, 0.66; 95% CI, 0.28-1.54) (Figure 3).

#### MACE

**Figure 4:**
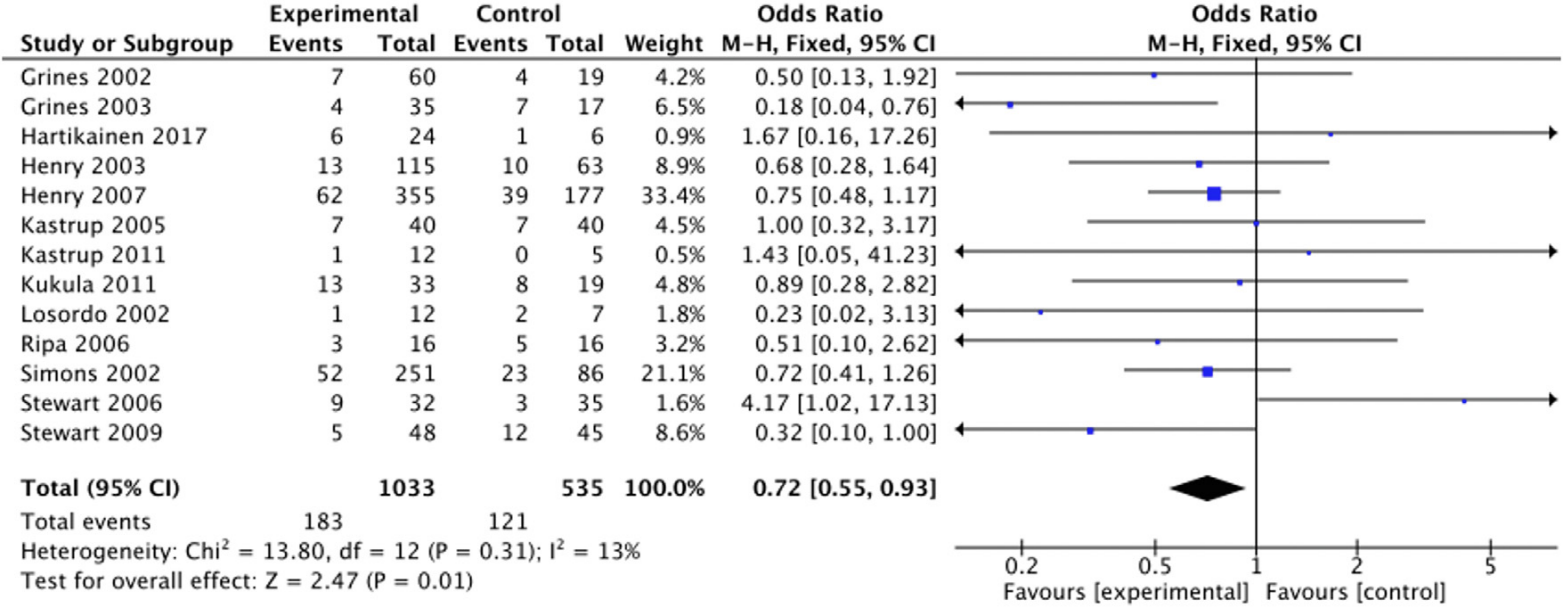
**Proangiogenic growth factor therapy and the risk of major adverse cardiovascular events.** Forest plot displays the odds ratio (OR) and 95% CI. df, degrees of freedom; M-H, Mantel-Haenszel.

All the trials reported MACE. Fourteen trials defined MACE as myocardial infarction, cardiac-related hospitalization and mortality. Two studies used different definitions: 1 used cardiac death or arrest, myocardial infarction, or stroke^22^; and 1 used myocardial infarction and unplanned hospitalization or revascularization due to myocardial ischemia.^21^ The analysis demonstrated a significant between-group difference: 22% (121/542) in the control group and 18% (183/1042) in the treatment group (OR, 0.72; 95% CI, 0.55-0.93) (Figure 4).

### Measures of anginal symptoms

#### Exercise tolerance

**Figure 5:**
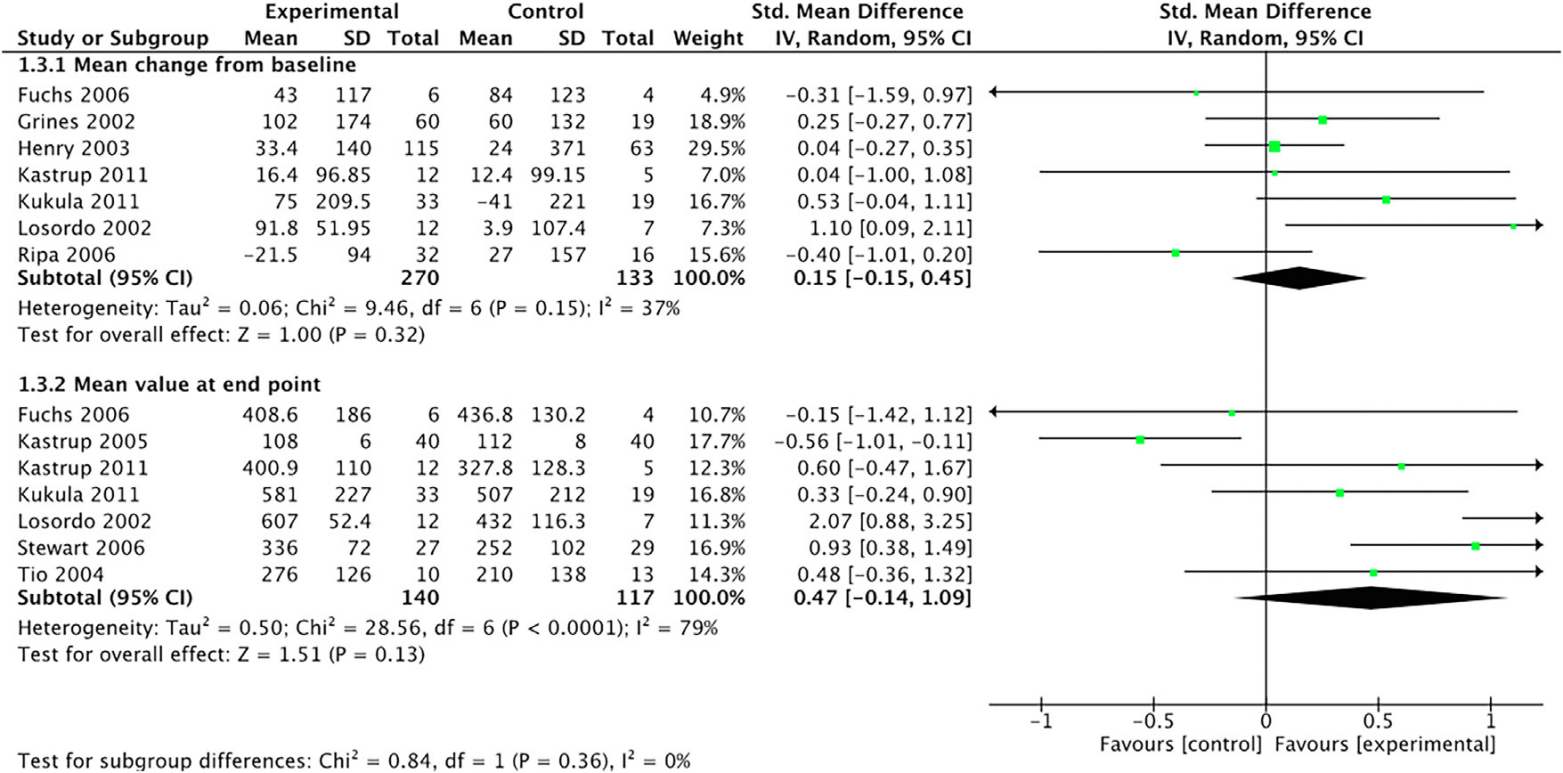
**Forest plot showing the standardized difference between the mean changes in exercise tolerance from baseline and mean value at the endpoint in patients who received proangiogenic growth factor therapy versus those who received maximal medical therapy.** Forest plot displays Standard Mean Difference (SMD) and 95% CI. df, degrees of freedom; M-H, Mantel-Haenszel.

A change in exercise tolerance was reported in all but 2 trials.^17^^,^^27^ Ten trials assessed exercise tolerance using a treadmill test with a modified Bruce,^22^^,^^24^^,^^25^^,^^29^^,^^31^ modified Balke,^18^^,^^21^ or an asymptomatic cardiac ischemia protocol.^19^^,^^28^^,^^30^ The remaining 4 used symptom-limited ergometers.^14^^,^^20^^,^^23^^,^^26^ The mean within-group change in exercise tolerance from baseline (SMD, 0.15; 95% CI, −0.15 to 0.45) and the mean value at the endpoint between the groups (SMD, 0.47; 95% CI, −0.14 to 1.09) demonstrated a nonsignificant improvement (Figure 5).

#### CCS class

**Figure 6:**
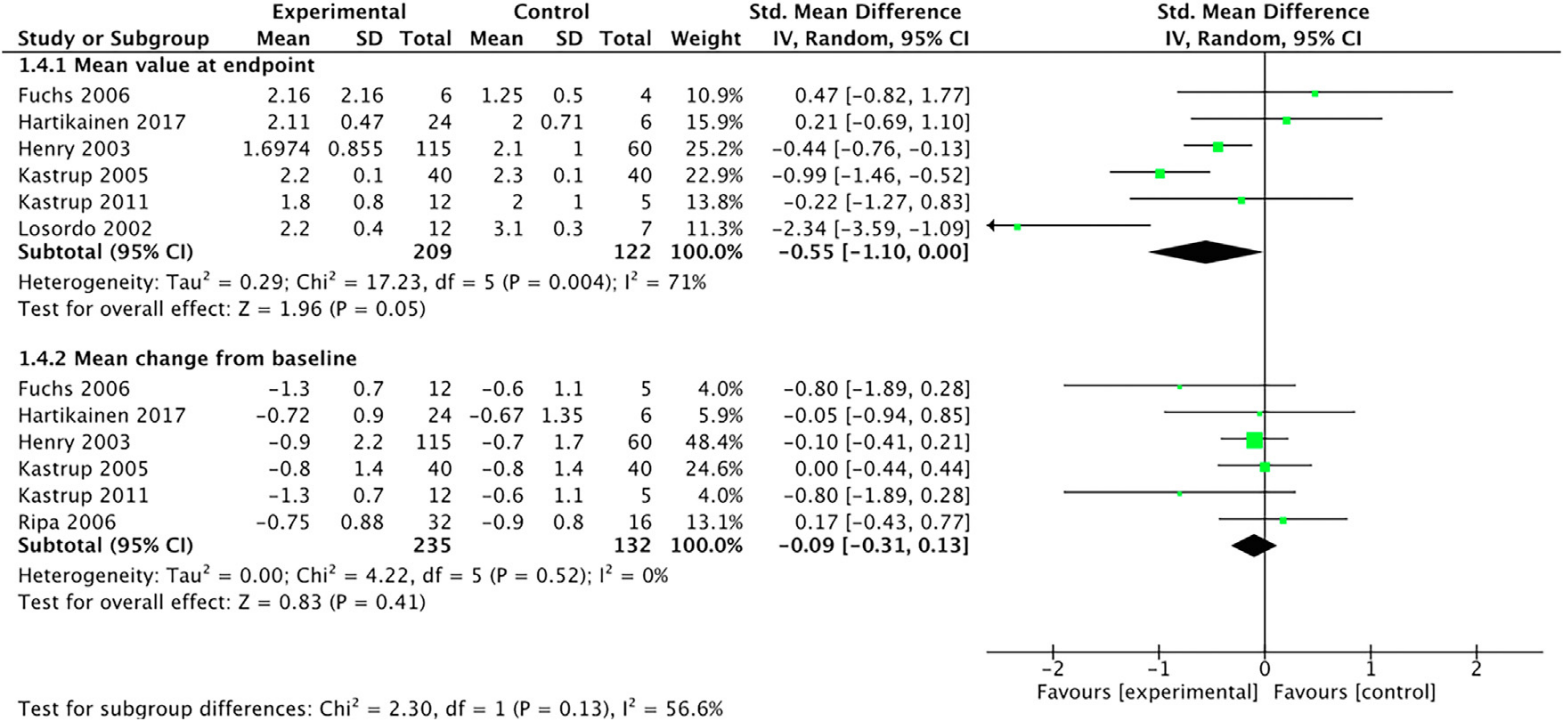
**Forest plot showing the standardized difference between the mean changes in Canadian Cardiovascular Society class from baseline and mean value at the endpoint in patients who received proangiogenic growth factor therapy versus those who received maximal medical therapy.** Forest plot displays standard mean difference (SMD) and 95% CI. df, degrees of freedom; M-H, Mantel-Haenszel.

Changes in CCS class were reported in all but 2 trials.^18^^,^^29^ The mean within-group change from the baseline demonstrated a nonsignificant improvement in the treated patients (SMD, −0.09; 95% CI, −0.31 to 0.13). However, significant improvements were demonstrated when the mean value at the endpoint was reviewed between the groups (SMD, −0.55; 95% CI, −1.10 to 0.00) (Figure 6).

#### Quality-of-life scores and the use of antianginal medications

Because the quality-of-life scores and the use of antianginal medications were not consistently reported, there were insufficient data to draw any conclusions.

#### Myocardial perfusion

Eleven trials used single-photon emission computed tomography (SPECT) scans as the imaging modality.^14^^,^^19^^,^^20^^,^^22^^,^^24^, ^25^, ^26^, ^27^^,^^29^, ^30^, ^31^ One trial also used a magnetic resonance imaging scan as a further perfusion endpoint.^14^ Of the remainder, 1 used stress echo,^18^ 2 used radiowater positron emission tomography (PET) scans,^17^^,^^23^ and 2 had no perfusion endpoint.^21^^,^^28^ All the assessments were made before treatment and at the end of follow-up. From the 11 that used SPECT, 10 reported no difference in the perfusion endpoint^14^^,^^19^^,^^20^^,^^22^^,^^24^, ^25^, ^26^, ^27^^,^^30^^,^^31^ and 1 small trial^29^ reported improved perfusion. There were no differences seen in the trial that used stress echo^18^; however, the 2 that used radiowater PET^17^^,^^23^ reported improvements in the myocardial perfusion reserve.

#### Safety

All the included trials evaluated the safety of proangiogenic growth factor therapy and reported that it was safe and well tolerated. The adverse events reported during follow-up included: mortality, MACE, congestive cardiac failure, stroke, arrhythmias, angina exacerbation, carcinoma, proliferative retinopathy, hepatitis, and fever. The adverse events were either not consistently reported or found not to differ between the treatment and control groups.

### Subgroup analyses

#### Delivery route

**Figure 7:**
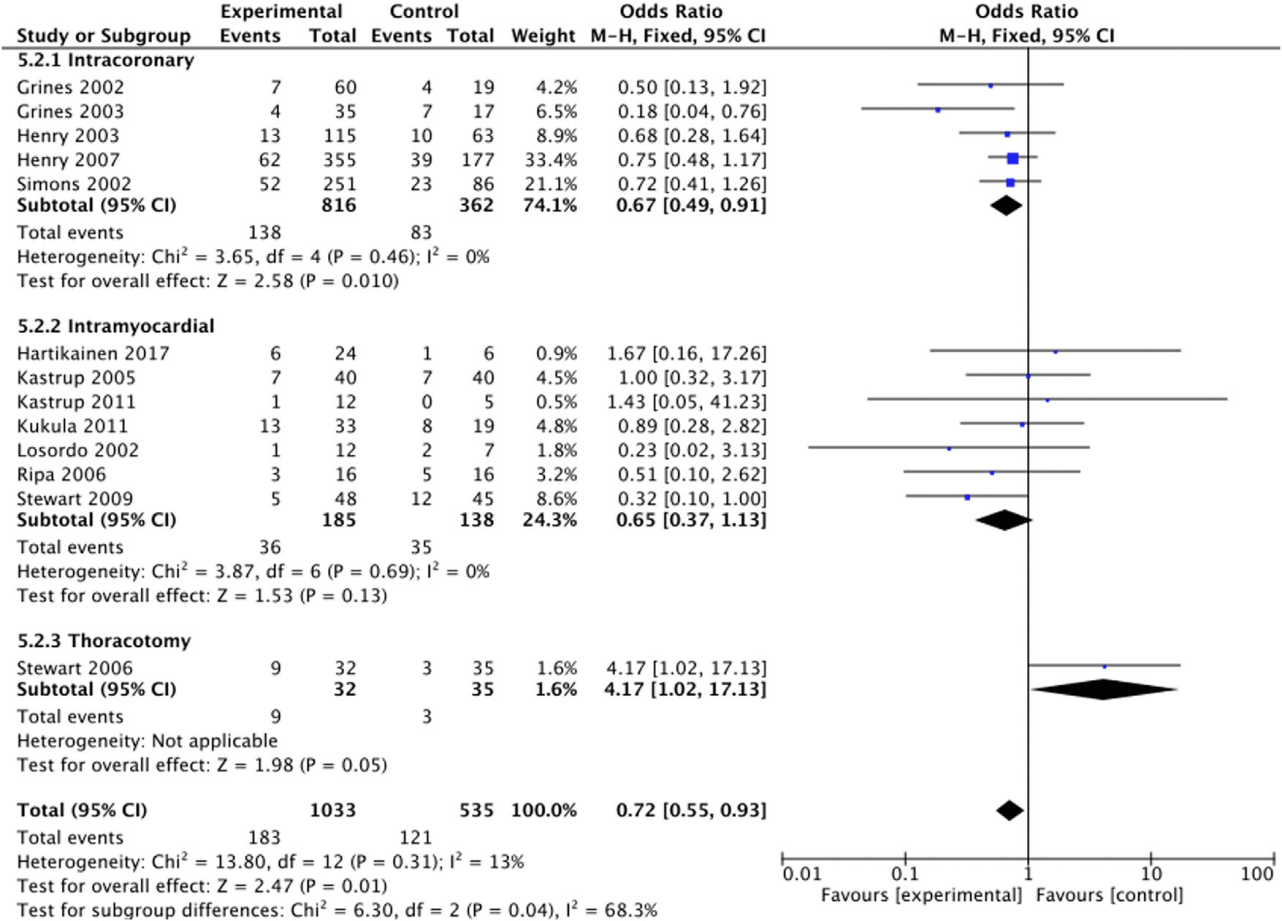
**Delivery route and risk of major adverse cardiovascular events.** Forest plot displays the odds ratio (OR) and 95% CI. df, degrees of freedom; M-H, Mantel-Haenszel.

**Figure 8:**
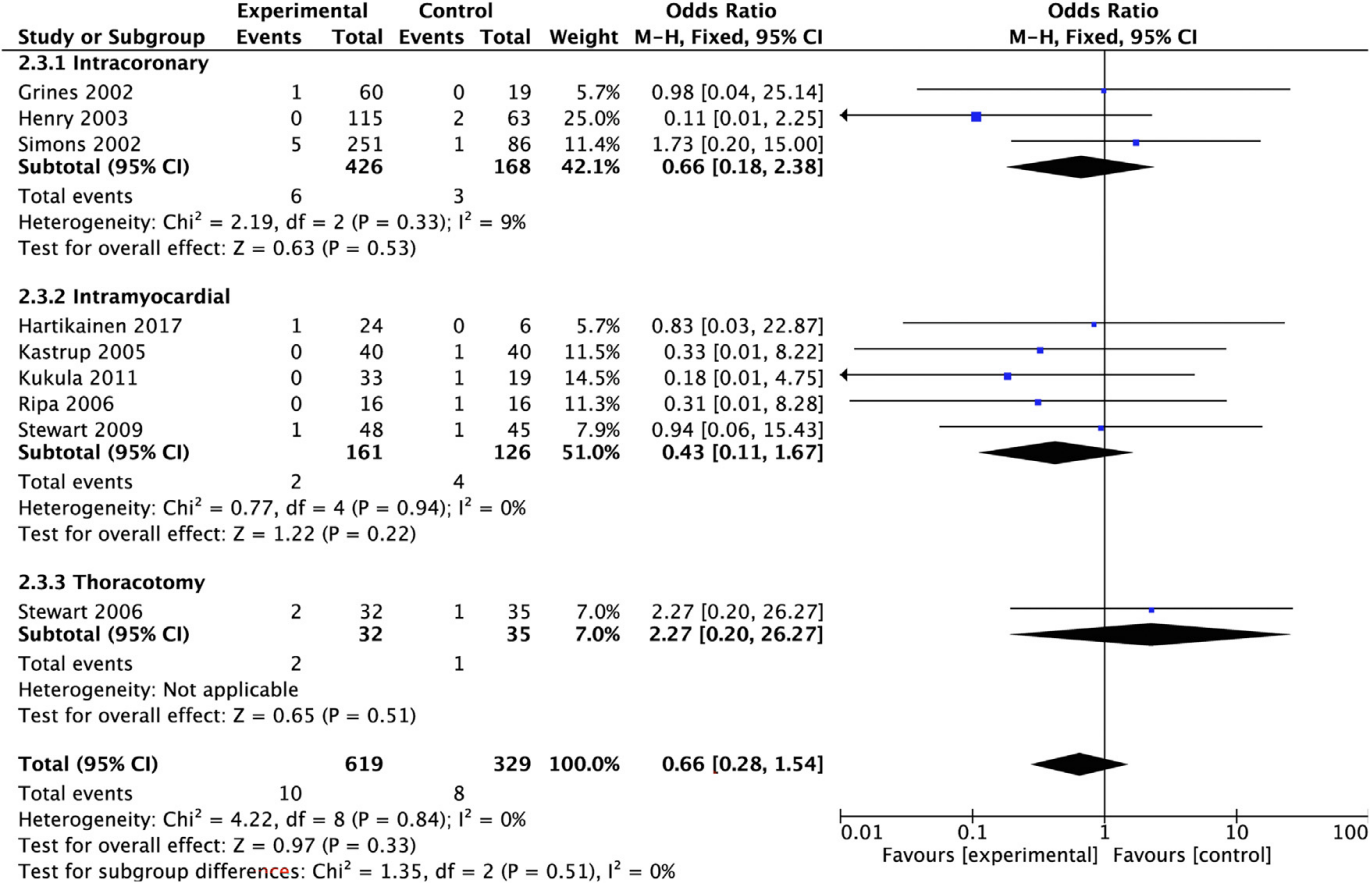
**Delivery route and risk of mortality.** Forest plot displays the summary the odds ratio (OR) and 95% CI. df. degrees of freedom; M-H. Mantel-Haenszel.

Five trials (n = 1178) used intracoronary delivery. MACE was 17% (138/816) in the treatment group and 23% (83/362) in the control group (Figure 7). Mortality was 1% in the treatment group (6/461) and 2% in the control group (3/185) (Figure 8). There were no reported periprocedural events.

Nine trials (n = 339) used transendocardial delivery. MACE was 19% (36/194) in the treatment group and 24% (35/145) in the control group (Figure 7). Mortality was 1% (2/194) in the treatment group and 3% (4/145) in the control group (Figure 8). Periprocedural MACE was 2% (8/339), including 1 procedural death related to pericardial effusion following the NOGA mapping procedure.

One study (n = 67) used minithoracotomy. MACE was 28% (9/32) in the treatment group and 9% (3/35) in the control group (Figure 7). Mortality was 6% (2/32) in the treatment group and 1% (1/35) in the control group (Figure 8). Periprocedural MACE was 8% (8/67).

Overall, no delivery route demonstrated a significant difference in all-cause mortality between the treatment and control arms. However, when the therapy was delivered via the intracoronary route, there was a significant reduction in MACE between the treatment and control arms (OR, 0.67; 95% CI, 0.49-0.91) (Figure 7).

#### Vector type

**Figure 9:**
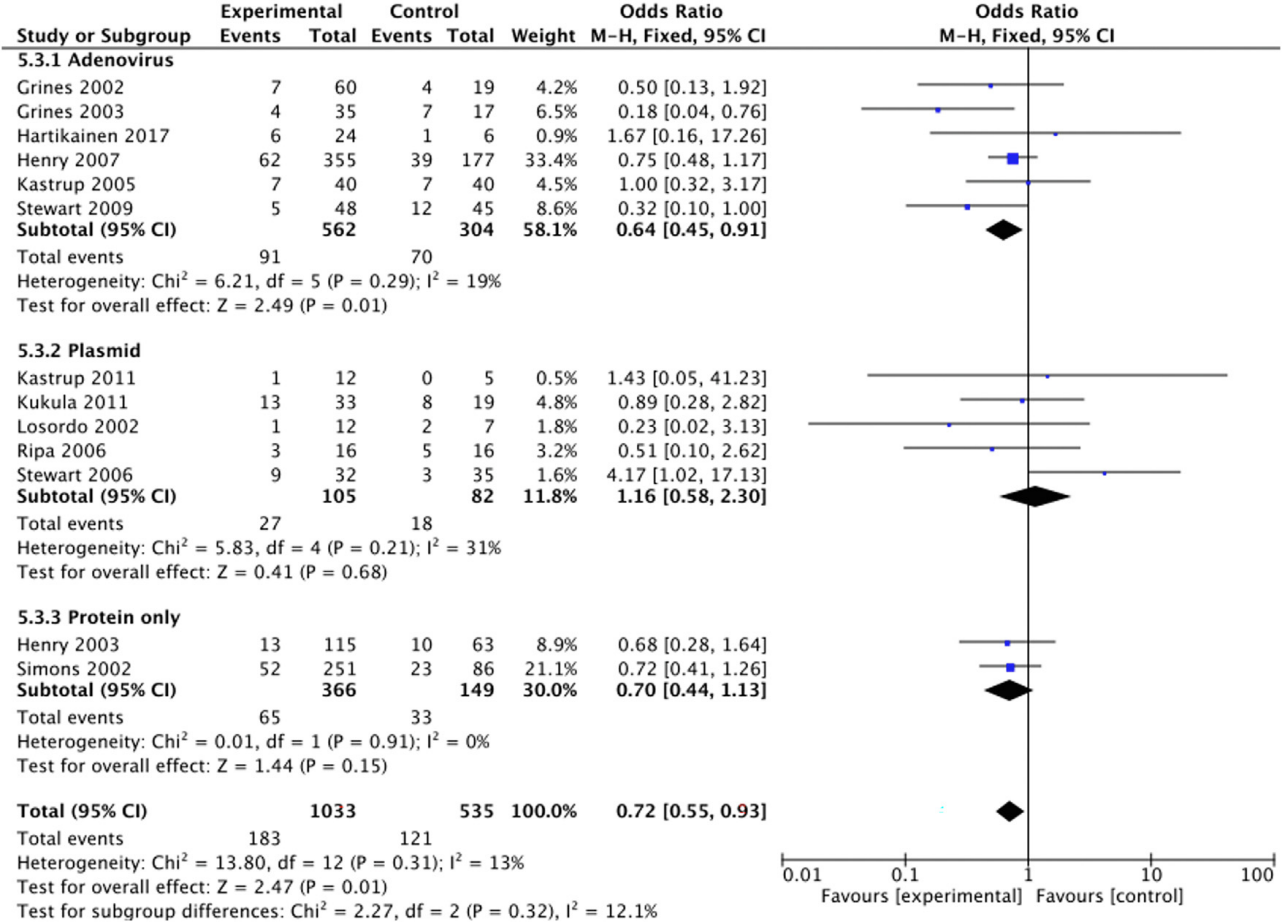
**Vector type and the risk of major adverse cardiovascular events.** Forest plot displays the odds ratio (OR) and 95% CI.

**Figure 10:**
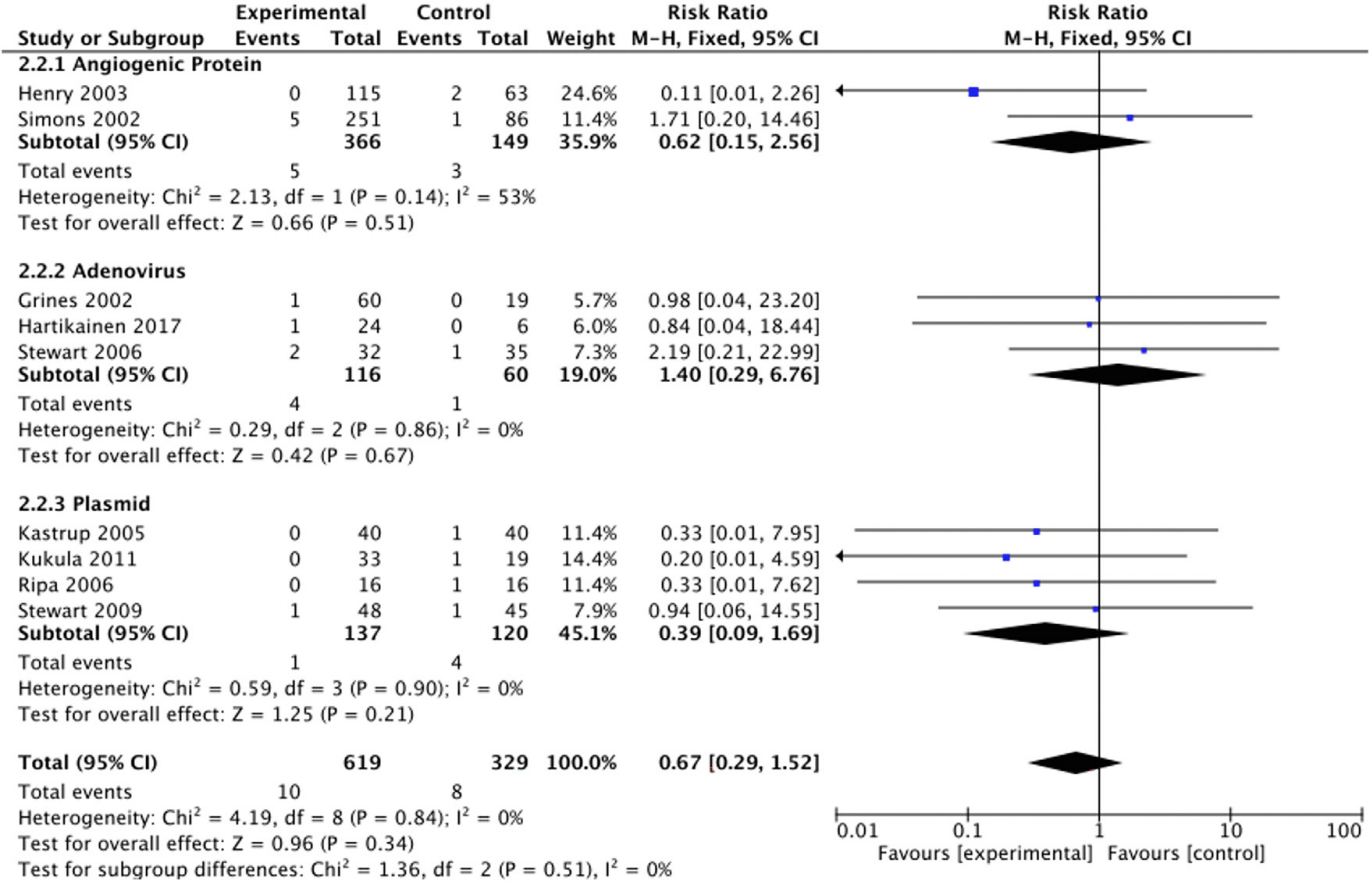
**Vector type and risk of mortality.** Forest plot displays the odds ratio (OR) and 95% CI.

Two trials (n = 515) delivered angiogenic proteins directly. Reported MACE was 18% (65/366) in the treatment group and 22% (33/149) in the control group (Figure 9). Mortality was 1% (5/366) in the treatment group and 2% (3/149) in the control group (Figure 10).

Fourteen trials (n = 1069) used gene therapy. Of these, 7 (n = 282) used a plasmid vector or control: MACE was 18% (27/152) in the treatment group and 14% (18/130) in the control group (Figure 9), and mortality was 1% (1/152) in the treatment group and 3% (4/130) in the control group (Figure 10). The remaining 7 (n = 866) used an adenoviral vector; there was a significant reduction in MACE between the treatment (16%; 91/562) and control groups (23%; 70/304) (OR, 0.64; 95% CI, 0.45-0.91; Figure 9). Mortality was 2% (4/169) in the treatment group and 1% (1/86) in the control group (Figure 10).

## Discussion

We presented the first meta-analysis of proangiogenic growth factor therapy for RFA (Central Illustration). Given the need for new evidence-based approaches for the treatment of RFA, this meta-analysis is important because the small sample sizes and different primary endpoints used in individual studies precluded seeing a clear efficacy signal.

**Central Illustration:**
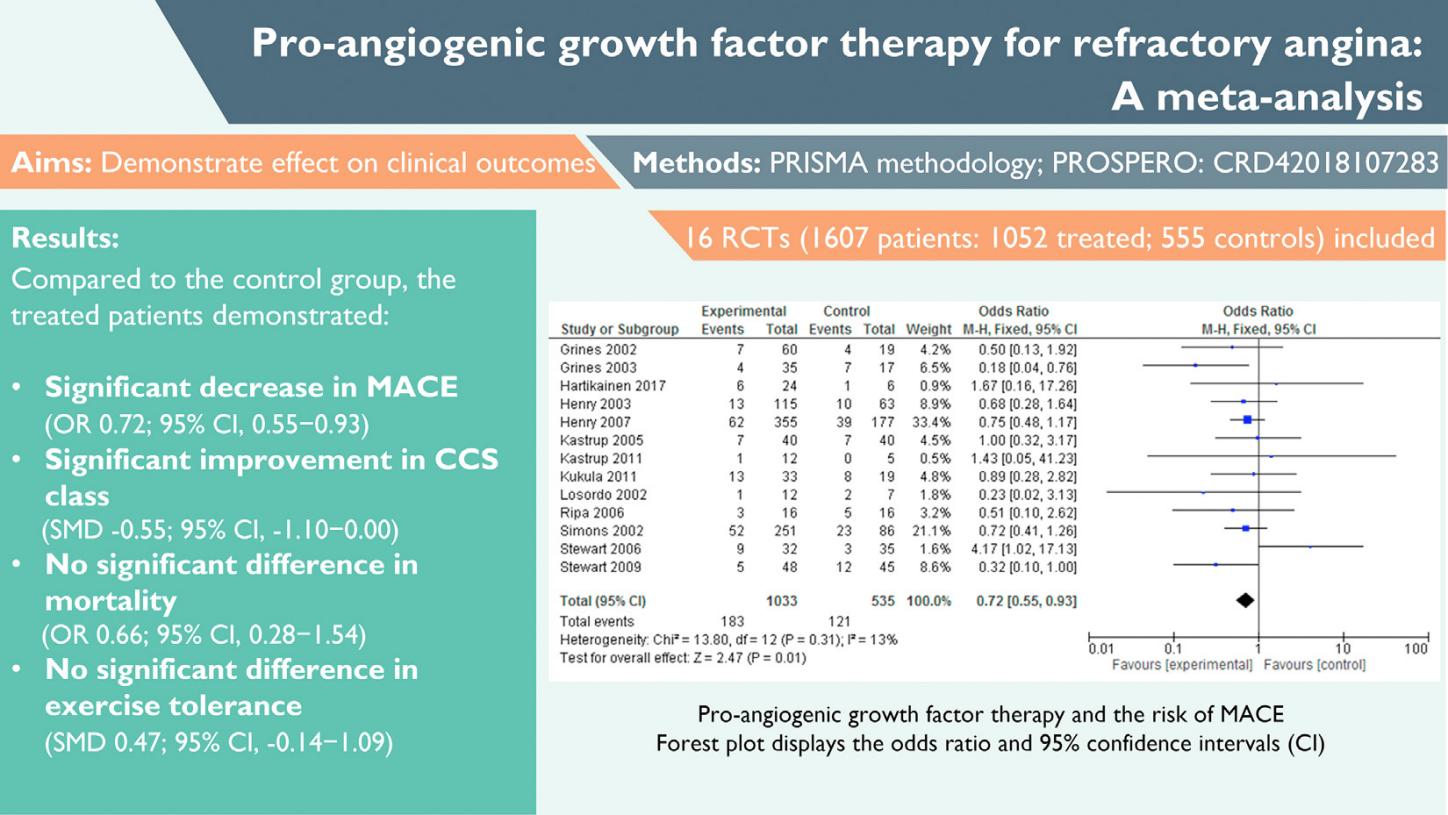
The main findings of this meta-analysis of proangiogenic growth factor therapy for refractory angina. CCS, Canadian Cardiovascular Society Angina Score; CI, confidence interval; MACE, major adverse cardiac event; OR, odds ratio; PRISMA, Preferred Reporting Items for Systematic Reviews and Meta-Analyses; RCT, randomized controlled trial; SMD, standard mean difference.

This meta-analysis demonstrated a significant improvement in MACE and CCS class in patients treated with proangiogenic growth factor therapy compared with that in the control group. However, no improvements were seen in mortality, which is likely because of the overall low event rate (2%, which is below the anticipated 3%-4% 1-year mortality in patients with chronic coronary syndromes^32^) and the variability in follow-up (3-24 months). Furthermore, 1 trial reported no mortality data^23^ and 1 trial combined MACE and mortality, which excluded them from the analysis.^21^ There were also no improvements seen in exercise tolerance. There were not enough reported data to evaluate the changes in the quality of life or use of medications. Interestingly, despite the improvements seen in MACE and CCS class, there were no improvements seen in myocardial perfusion in the majority of the trials (except the 2 that used PET imaging). This is unexpected given the proposition that angiogenic growth factors improve blood supply by stimulating angiogenesis. However, the fact that trials using PET imaging did demonstrate an improvement in myocardial perfusion may well reflect the relative sensitivity of this technique compared with those that used SPECT (especially given the limitations of SPECT in pixel size and the subsequent difficulty in detecting diffuse ischemia).

Importantly, proangiogenic growth factor therapy (delivered either as recombinant proteins or gene therapy) was shown to be safe, with a periprocedural complication rate of 2%, which is comparable with that of percutaneous coronary intervention.^33^ The biggest potential concern regarding proangiogenic growth factor therapy has been new cases of cancer; however, 8 and 10 year follow-up data have shown no evidence of a higher incidence of cancer^34^, ^35^ (although the sample size remained relatively small).

Our analysis included trials that used proteins from both the VEGF and FGF families; however, given the small sample size, it is difficult to draw conclusions regarding their comparable efficacy.

Fourteen trials used genes to deliver proangiogenic growth factor therapy to the cell’s nucleus via a vector, which was either viral or nonviral mediated. The 2 gene therapy vectors that have been tested in the treatment of RFA are plasmid DNA and adenoviral vectors. Plasmid vectors have the therapeutic gene directly incorporated into a circular DNA strand; they are cheaper, accessible to mass manufacture, and have little or no immunogenicity, which allows for repeated dosing. Adenoviral vectors are more complicated to manufacture, and there is an associated risk of viral toxicity (which is mitigated by ensuring that the virus is replication incompetent). However, adenoviral vectors are more efficient in infecting cells and transferring genetic material than nonviral vectors.^36^ This meta-analysis suggests there is no significant difference between either vector in terms of MACE or mortality. Although there was a stronger signal with plasmid delivery, it may simply reflect the small sample size (7 trials, n = 282, used plasmid and 7 trials, n = 787, used adenoviral vectors). Different dose levels were not analyzed because it was assumed that appropriate levels for therapy were used.

Regarding delivery, studies that explored 3 routes were included in this meta-analysis: transepicardial (via minithoracotomy), transendocardial, and intracoronary. Although the transepicardial approach allows direct myocardial access (and, hence, a potentially more efficient delivery), it is associated with problems related to major surgery. The percutaneous transendocardial approach is less invasive, and electromechanical mapping systems enable myocardial targeting.^37^ Theoretically, one may expect that intramyocardial delivery facilitates higher concentrations of the therapeutic product alongside longer-lasting biological effects in the myocardium compared with an intracoronary delivery.

The intracoronary route is easier and has a lower complication rate^37^ compared with the abovementioned intramyocardial methods. Some preclinical data have suggested that it is a less efficient route to deliver viral vectors,^38^^,^^39^ whereas others have noted that, despite lower retention rates, these less invasive techniques (particularly when paired with proximal balloon occlusion to limit competitive flow) result in an efficient uptake of both adeno-associated virus and similarly sized nanoparticles.^40^

Our meta-analysis demonstrated a significant reduction in MACE when the therapy was administered via the intracoronary route (n = 1178), which was not seen in the transendocardial group (n = 339) – although this may be because of the different patient population sizes. The potential, theoretical therapeutic benefits of the intramyocardial approach should also be weighed against its potential for a higher complication rate: Our analysis demonstrated a periprocedural adverse event rate of 2% versus none with the intracoronary route. Minithoracotomy (n = 67), although it carries theoretical targeting benefits, was also associated with higher mortality and MACE rates, suggesting that percutaneous delivery methods are preferential overall. Caution is required, however, when interpreting these comparisons between delivery routes because of the large sample size differences.

Further trials of proangiogenic growth factor for RFA have been planned or are ongoing. The EXACT trial, a single-arm, dose escalation phase I/II study investigating a novel adenoviral vector that utilizes multiple isoforms of VEGF, is currently recruiting,^41^ The ongoing pan-European phase II trial, ReGenHeart (NCT03039751), was designed after the KAT301 trial^17^ and is investigating the intramyocardial delivery of VEGF-D. A phase III trial has been planned based on the findings of the AGENT trial series to investigate the use of Ad5FGF-4 in 300 female patients, but it is not yet recruiting.

### Limitations

There are limitations to consider while interpreting our findings. We standardized the definitions of the endpoints between individual trials to avoid heterogeneity, with no individual study or trial being powered to assess benefits in terms of MACE or mortality. However, the differences in the definitions were modest and are unlikely to have affected the overall outcome. Although attempts were made to contact the authors regarding missing data (particularly regarding sequence generation and allocation concealment), its impact on the overall effect and potential bias should be acknowledged. Because this analysis did not have access to individual patient data, no temporal information with respect to events is available. It should also be noted that the trials had different durations (ranging from 3 to 24 months). Overall, the results (including any differences or lack thereof) should be considered with caution given the overall small number of events and patients. Thus, we also cannot draw a firm conclusion regarding the optimal delivery method, vector type, and protein; the subgroup analyses should only be considered exploratory. Finally, as with other meta-analyses, the ability to draw valid conclusions is limited by small study biases, which likely inflated the measures of efficacy.

## Conclusion

Our meta-analysis demonstrates that proangiogenic growth factor therapy for patients with RFA is safe and leads to improvements in MACE and CCS class. Given the need for further therapeutic options for the treatment of patients with RFA, larger studies are justified to look for a definitive beneficial signal and ensure that the optimal delivery route, delivery vector and protein type are identified.

## Declaration of competing interest

The authors declared no potential conflicts of interest with respect to the research, authorship, and/or publication of this article.

## Funding sources

This research did not receive any specific grant from funding agencies in the public, commercial, or not-for-profit sectors.

## Ethics statement and Patient Consent

Ethical or institutional review board approval was not required because this was a meta-analysis of existing clinical trials that all had independent approved ethical review.
