# Supplementary material for: Proangiogenic Growth Factor Therapy for the Treatment of Refractory Angina: A Meta-analysis
Source: J Soc Cardiovasc Angiogr Interv. 2023 Jan 2;2(1):100527. doi: 10.1016/j.jscai.2022.100527 (PMC11307391; doi:10.1016/j.jscai.2022.100527)
Supplement: Supplementary Appendix [file mmc1.docx]

**Supplemental Appendix**

| **Online Table 1: Characteristics of included studies** | **2** |
| --- | --- |
| **Data Supplement** | **7** |
|  |  |

**Supplementary Figure 1 9**

**Online Table 1:** Characteristics of included studies

| Study; (Country, trial name) | Study Design | (n) | Vector | Inclusion Criteria | Exclusion Criteria | Cell Type | Delivery Route | Outcome | Follow up |
| --- | --- | --- | --- | --- | --- | --- | --- | --- | --- |
| ***Recombinant Proteins*** | | | | | | | | | |
| Simons et al^21^, 2002  (USA, FIRST)  [angiogenic protein] | Randomised, placebo controlled trial, double blind | 337 | N/A | CCS III-IV; ETT 3-13mins; Inducible ischemia on SPECT; EF≥30%; | Unstable angina; MI; CABG or PCI within 3 months; malignancy 10 years; retinopathy or renal dysfunction | rFGF2 | IC | 1: ETT (90 Days)  2: ETT (180); CCS; Qol – SAQ/SF-36; SPECT ischaemia segments | 6 months |
| Henry et al^28^, 2003  (USA, VIVA)  [angiogenic protein] | Randomised, placebo controlled trial, double blind | 178 | N/A | 40 to 75years; stable angina; No further revasc options; perfusion defect; ETT between 3 and 11 mins; | ACS; CABG within 6 months; PCI 3 months; previous rx for RFA; EF <25%; pregnancy; hx of cancer within 5 years; proliferative retinopathy; or macular degeneration; renal insufficiency or severe concurrent illness | rhVEGF | IC + IV | 1: Change in ETT 2 months  2: ETT 120 days  Rest and stress MPS day 60; angina class; QoL | 4 months |
| ***Gene Therapy: 5FGF-4*** | | | | | | | | | |
| Grines et al^14^ 2002  (USA, AGENT) | Randomised, placebo controlled trial, double blind | 79 | Ad | 30-75 years; CCS II/III; ETT >3mins; Angina on exercise; 1 prox vessel had <70% stenosis; | LMS stenosis >50%; coronary ostial stenosis; EF <40%; patent bypass grafts; CABG <1 year, PCI< 6 months; unstable angina; NYHA 3 or 4; LBBB; mobitz block type II or complete heart block; AF or ventricular arrhythmia; child bearing potential; diabetic retinopathy; malignancy; creat cl <45; | Ad5FGF-4 | IC | 1: Safety  2: ETT; Time to angina | 12 months |
| Grines et al^24^, 2003  (USA, AGENT-2) | Randomised, placebo-controlled trial, double-blind | 52 | Ad | 30-75 yrs; CCS II-IV; OMT; No further revasc options; EF>30% on SPECT; | >70% stenosis in LMS; CABG within 6 months; PCI within 4 months; MI <6weeks; NYHA 4; Persistent AF or Flutter; HIV +VE; Chronic immunosuppressive therapy; hepatic disease; T1DM with proliferative retinopathy; malignant tumour childbearing potential; abciximab within 30 days; adenosine contraindicated | Ad5FGF-4 | IC | 1: Perfusion defect decrease  2: Safety | 12 months |
| Henry et al^17^, 2007  (USA, AGENT-3/4) | A randomized, parallel group, double-blind, placebo-controlled | 532 | Ad | CCS class II-IV; 30-75 years; OMT; EF ≥30%; 1-,2- or 3-vessel CAD with one prox stenosis <70; ET 3-10 mins; No further revasc options | Unstable angina, illness, untreated ventricular arrhythmias, ECG unable to show myocardial ischaemia; illness resulting in being unable to perform ETT; LMS stenosis >70%; CABG <6 months; PCI <8 weeks; NYHA 3/4; non-proliferative or proliferative retinopathy; macular oedema/photocoagulation; immunosuppressive therapy; women of childbearing potential | Ad5FGF-4 | IC | 1: ETT  2: Time to 1mm ST-depression on ETT; Time to angina onset on ETT; Change in CCS class; Pts with ≥30% variability in ETT; Coronary events or death 1 year; Angina incidence; NTG use; QoL | 24 months |
| ***Gene Therapy: VEGF*** | | | | | | | | | |
| Vale et al^26^, 2001  (USA) | Randomized single-blind placebo controlled trial | 6 | Pl | CCS II/IV; OMT; Rev ischaemia on SPECT | EF<20%; malignancy; diabetic retinopathy; | phVEGF-2 | TEC | 1: Safety and feasibility  2: SPECT; ETT; Angina episodes; GTN consumption; LVEF | 12 months |
| Losordo et al^22^, 2002 (USA) | Randomized double-blind placebo controlled trial | 19 | Pl | CCS III/IV; OMT; No revasc options; rev isch on SPECT; | Mailgnancy; diabetic retinopathy; EF<20%; | phVEGF-2 | TEC | 1: CCS class, ETT  2: SAQ  Angina episodes  GTN usage | 3 months |
| Fuchs et al^25^, 2006  (Israel) | Randomized, double-blind, placebo-controlled trial | 10 | Ad | 18-80 years; CCS III-IV; OMT; No further revascularization options; ETT between 90 secs – 8mins with 1mm ST depression | ACS/CVA within 4 weeks; LV thrombus; Unprotected LMS/LAD; uncontrolled HTN or Hypotension; ICD; EF <25%; significant VHD | AdVEGF_121_ | TEC | 1: Safety and Feasibility  2: ETT; CCS class; SAQ | 12 months |
| ***Gene Therapy: VEGF-A*** | | | | | | | | | |
| Tio et al, 2004^20^  (Netherlands) | Randomised, controlled trial | 23 | Pl | >18 years; CCS≥3; symptoms despite OMT; >15% myocardium ischaemia on PET | pregnancy, lactation, history of cancer, proliferative eye disease; severe concurrent illness EF <20% significant aortic stenosis | VEGF-A_165_ | TEC | 1.PET – myocardial ischaemia  2. CCS, ETT | 3 months |
| Kastrup et al^16^,2005  (Denmark, EuroinjectOne) | Randomised, placebo-controlled trial, double-blind phase II | 80 | Pl | 18-75 years; CCS≥3; symptoms despite OMT; No further revasc options | EF<40%; ACS within 3 months; DM with proliferative retinopathy; potential malignancy; chronic inflammatory disease; pre-menopausal women | VEGF-A_165_ | TEC | 1: SPECT – myocardial perfusion defects  2:  Safety; Change in wall motion; CCS; Angina frequency; GTN consumption; SAQ; Exercise capacity | 3 months |
| Ripa et al^18^, 2006 (Denmark, NEUOPEGEN) | Phase 1 safety and efficacy study | 32 | Pl | 18 – 75 years; Significant reversible ischaemia on SPECT; One remaining large coronary vessel from which new collaterals could be formed; CCS III-IV; | ACS within 3 months; diabetes with proliferative retinopathy; diagnosed or suspected cancer; chronic inflammatory disease; fertile women | VEGF-A_165_  + G-CSF | TEC | 1: Change in SPECT perfusion  2: CCS class  SAQ  Freq of angina attacks  GTN consumption  Exercise capacity  LV volume by SPECT/MRI | 3 months |
| Stewart et al^27^,2009  (Canada, NORTHERN) | Randomised, placebo-controlled trial, double-blind phase II | 93 | Pl | CCS 3/4; OMT; No further Revasc options; Rev isch on SPECT/ Viable myocardium; EF≥20%; ≤75yrs; LV wall ≥0.9cm; | Too unwell to undergo procedure; Too high risk for growth factor; a significant proximal lesion | VEGF-A_165_ | TEC | 1: Change in SPECT  2: ETT; Troponin; CCS class | 6 months |
| Kukula et al^19^, 2011  (Poland, VIF-CAD) | Randomised, placebo-controlled trial, double-blind | 52 | Pl | CCS III-IV; OMT; No further revascularisation options; Perfusion defect on SPECT; EF>35% | ACS within 4 weeks; diabetes with proliferative retinopathy; diagnosed or suspected malignancy or < 10 years from successfully treated malignant disease; chronic inflammatory or autoimmune disease | phVEGF-A_165_/bFGF | TEC | 1: SPECT perfusion change  2: CCS Class; ETT  MACE; Collateral circulation; LVEF  FGF/VEGF expression; QoL | 12 months |
| Stewart et al^15^, 2006  (Canada, REVASC) | Randomised, placebo-controlled trial, phase II | 67 | Ad | 18-80 years; CCS II-IV; OMT; No Revasc options; significant stenosis in coronary angiography; Ability to perform ETT between 90s – 8 mins | Pregnancy; ACS/CVA within 2 weeks; LV thrombus; 90% lesion in LMS/prox LAD; electrical activity which interferes with ECG; uncontrolled HTN/Hypotension; ICD or PPM; Severe CHF; LVEF <25%; VHD; planned Heart Transplant; significant hematological abnormalities; anticoagulation; unable to perform ETT; malignancy; immunocompromised; renal or hepatic dysfunction; proliferative retinopathy | AdVEGF-A_121_ | Mini-thoracotomy | 1: ETT – time to ST depression  2: TET; Mean CCS class; SAQ; SPECT (in 50 pts) | 6 Months |
| Kastrup et al^23^ 2011  (Denmark, NOVA) | Randomised, placebo-controlled trial, double-blind phase II | 17 | Ad | 18-80 years; CCS II-IV; OMT; No Revasc options; significant stenosis in coronary angiography; TET (bicycle) between 2-8mins; | ACS in the last 6 weeks; EF <25%; NYHA 3-4; DM with proliferative retinopathy; suspected cancer; chronic inflammatory disease; fertile women | AdVEGF-A_121_ | TEC | 1: ETT  2: Reversible perfusion defect (SPECT); Total exercise duration; Time to ST depression on ETT; CCS class; Angina Frequency; NTG use | 12 months |
| ***Gene Therapy: VEGF-D*** | | | | | | | | | |
| Hartikainen et al^13^ 2017  (Finland, KAT 301) | Randomised, placebo-controlled trial, double-blind phase I/IIa | 30 | Ad | 30 to 80 years; CCS II-III on OMT; angina or ischemic ST-depression (≥1mm) in ET; significant stenosis in coronary angiography; contraindication to PCI or CABG; LV wall >8mm on TTE | T1DM or diabetic retinopathy/nephropathy, anaemia, leukopenia, leukocytosis, thrombocytopenia, CKD, liver insufficiency, haematuria of unknown origin, severe hypertension, significant hypotension, BMI >35, acute infection, immunosuppressive and EF <25% | VEGF-D^∆N∆C^ | TEC | 1: Safety  2: PET perfusion; LVEF; CCS Class; QoL; Hospitalisations;  Medications | 12 months |
| **Abbreviations:** IC, Intracoronary; TEC, transendocardial; CCS, Canadian cardiovascular society functional class; ETT, Exercise Tolerance; MPS, Myocardial Perfusion Scan; LVEF/EF, Left ventricular Ejection Fraction; MI, Myocardial Infarction; CABG, Coronary Artery Bypass Graft; PCI, Percutaneous Coronary Intervention; QoL, Quality of Life; SAQ, Seattle Angina Questionnaire; SF-36, San Francisco 36 score; SPECT, Single photon emission CT scan; ACS, Acute Coronary Syndrome; LMS, Left Main Stem; NYHA, New York Heart Association functional class; AF, Atrial Fibrillation; LBBB, Left Bundle Branch Block; T1DM, Type 1 Diabetes Mellitus; CAD, Coronary Artery Disease; NTG, Nitroglycerine; CVA, Cerebrovascular Accident; OMT, Optimal medical therapy; VHD, Valvular Heart Disease; LAD, Left Anterior Descending; CHF, congestive heart failure; DM, Diabetes Mellitus; TTE, Transthoracic Echo; BMI Body Mass Index; MACE, Major Adverse Cardiovascular Outcomes; CKD, Chronic Kidney Disease; HTN, Hypertension; USA, United States; LV, Left Ventricular | | | | | | | | | |

**Data Supplement**

Medline search strategy:

46           Medline               (coronary arter* disease* OR angina OR chronic symptomatic coronary arter* disease* OR chronic myocardial isch?em*).ti,ab          148062

48           Medline               exp "MYOCARDIAL ISCHEMIA"/ OR exp "ANGINA PECTORIS"/    404637

49           Medline               exp "CORONARY ARTERY DISEASE"/        54428

50           Medline               (48 OR 49)           404637

51           Medline               exp "CHRONIC DISEASE"/             249974

52           Medline               (chronic* OR refractory OR intractable OR drug resistant).ti,ab   1264006

53           Medline               (51 OR 52)           1326781

54           Medline               (50 AND 53)        27224

55           Medline               (46 OR 54)           163274

56           Medline               exp "ANGIOGENIC PROTEINS"/ 54338

57           Medline               ((angiogenic ADJ protein*) OR angiogenesis).ti,ab            82250

58           Medline               exp "INTERCELLULAR SIGNALING PEPTIDES AND PROTEINS"/ad                36431

59           Medline               (56 OR 57 OR 58)               151321

60           Medline               exp "GENETIC THERAPY"/            45582

61           Medline               (gene* ADJ therap*).ti,ab           46499

62           Medline               (60 OR 61)           67570

63           Medline               (59 OR 62)           214723

64           Medline               (55 AND 63)        1952

65           Medline               (55 AND 63)  [Document type Clinical Study OR Clinical Trial OR Clinical Trial, Phase I OR Clinical Trial, Phase Ii OR Clinical Trial, Phase Iii OR Clinical Trial, Phase Iv OR Comparative Study OR Controlled Clinical Trial OR Meta-analysis OR Multicenter Study OR Randomized Controlled Trial OR Review OR Twin Study OR Validation Studies]               854

Cochrane Search strategy:

[duplicated the medline search strategy above, but without any limitations]

Embase search strategy:

1     (coronary arter* disease* or angina or chronic symptomatic coronary arter* disease* or chronic myocardial isch?em*).ab,ti.

2     exp *coronary artery disease/

3     exp *heart muscle ischemia/

4     exp *angina pectoris/

5     2 or 3 or 4

6     exp *chronic disease/

7     (chronic* or refractory or intractable or drug resistant).ab,ti.

8     6 or 7

9     5 and 8

10     1 or 9

11     exp *angiogenic protein/

12     exp *"peptides and proteins"/dt

13     ((angiogenic adj protein*) or angiogenesis).ab,ti.

14     11 or 12 or 13

15     (gene* adj therap*).ab,ti.

16     exp *gene therapy/

17     15 or 16

18     14 or 17

19     10 and 18

20     limit 19 to (clinical trial or randomized controlled trial or controlled clinical trial or multicenter study or phase 1 clinical trial or phase 2 clinical trial or phase 3 clinical trial or phase 4 clinical trial)

21     limit 20 to (meta-analysis or "systematic review")

**Supplementary Figure 1.** Funnel plots showing risk of publication bias for MACE (A) and mortality (B).

The symmetrical distribution for both MACE and mortality plots suggest a low risk of publication bias.


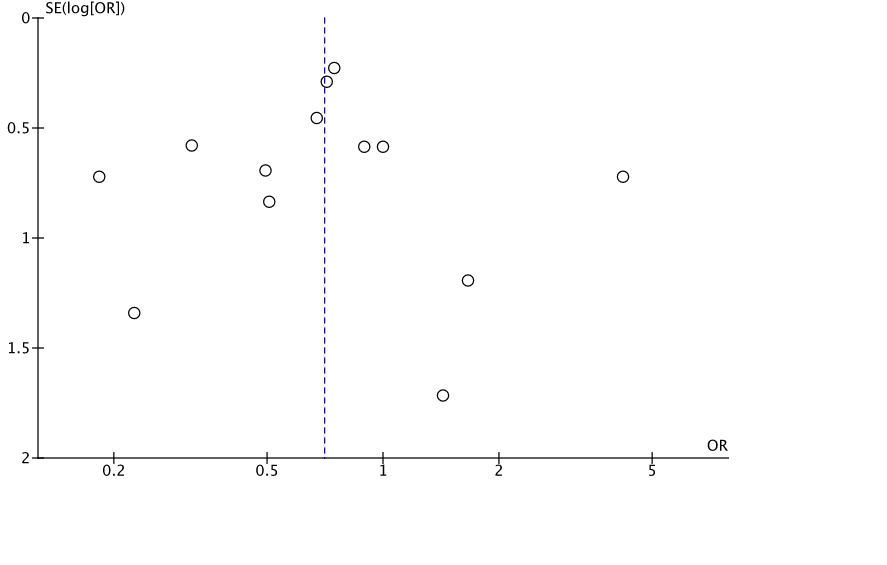


**A**


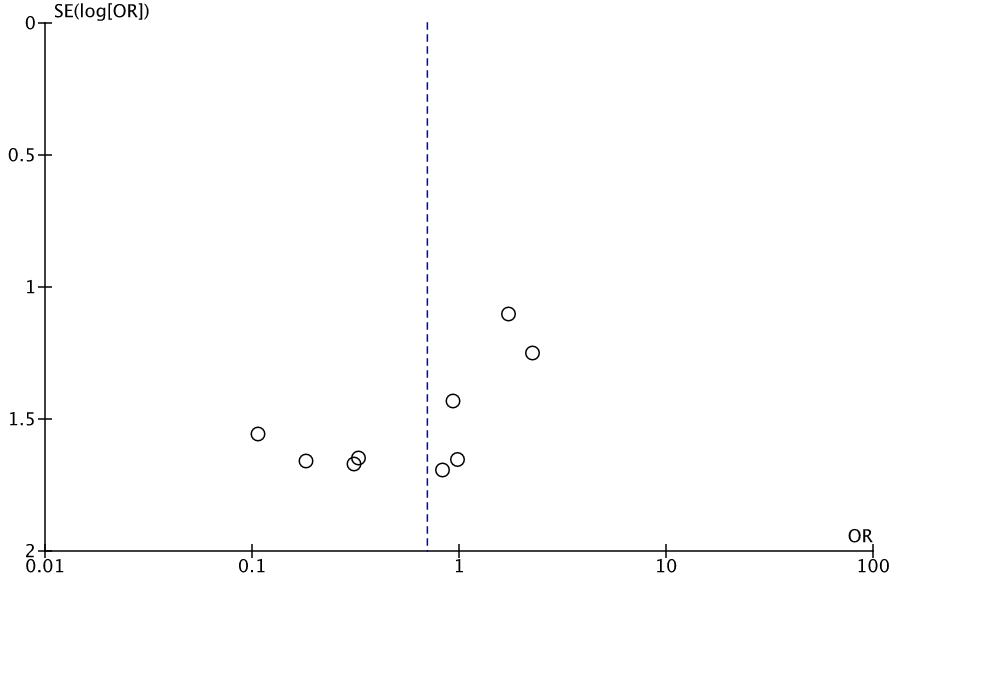


**B**
